# Thin Slice Unenhanced Brain CT Can Detect Aneurysms Larger than 7 mm

**DOI:** 10.5334/jbsr.2749

**Published:** 2022-04-26

**Authors:** Ludger Feyen, Patrick Haage, Patrick Freyhardt, Peter Schott, Marcus Katoh, Christian Blockhaus, Katharina Melber

**Affiliations:** 1 Helios Klinikum Krefeld, DE; 2 University Witten/Herdecke, Faculty of Health, School of Medicine, Alfred-Herrhausen-Straße 50, 58448 Witten, DE; 3 Diagnostic and Interventional Radiology, HELIOS University Hospital Wuppertal, University Witten/Herdecke, Germany, Heusnerstraße 40, 42283 Wuppertal, DE; 4 Helios Klinikum Wuppertal, DE; 5 Heart Centre Niederrhein, Department of Cardiology, Helios Clinic Krefeld, DE; 6 Sana Klinikum Duisburg, DE

**Keywords:** intracranial aneurysm, unruptured intracranial aneurysm, NECT, subarachnoid hemorrhage, Phases score

## Abstract

**Purpose:**

To evaluate the detection rate of intracranial aneurysms on reconstructed thin slice non enhanced CT (NECT) scans.

**Methods:**

NECT scans from 34 patients with 35 aneurysms and 35 individuals without aneurysms were collected. Thin slice maximum intensity projections of the NECT scans were reconstructed. One observer evaluated the native images twice with a time interval of six month between both passes with respect to the prevalence and location of an aneurysm. The size and location of the aneurysms were evaluated in corresponding CT-Angiography and Time of flight datasets. A logit regression analysis was performed with size and location as dependent variables. 2 × 2 tables were constructed. The sensitivity and false negative rate were calculated for aneurysms with 0–6.9 mm, 7–9.9 mm and 10–20 mm and the under the curve (AUC) was calculated.

**Results:**

The overall detection rate of the aneurysms was 63% for the first pass and 66% for the second pass in the reconstructed NECT scans. The detection rate of aneurysms is size dependent. The sensitivity to detect aneurysms with a size of 0–6.9 mm was 0.09 and 0.03, for aneurysms with a size of 7–9.9. mm was 0.8 and 0.7 and for aneurysms with a size of 10–20 mm was 0.92 for both passes.

The AUC was 0.77 for the first pass and 0.78 for the second pass.

**Conclusions:**

NECT scans can be used to detect a significant proportion of intracranial aneurysms larger than 7 mm if properly displayed and reconstructed. These patients should receive further vascular imaging to prevent future aneurysm related subarachnoid hemorrhage.

## Introduction

The prevalence of unruptured intracranial aneurysms is estimated at around 3.2% in the general population [1]. The incidence of aneurysm related subarachnoid hemorrhage is averaged at 6 per 100,000 per year in Europe with a mortality of 35% [2,3].

The risk of rupture of a cerebral aneurysm is size and location dependent. Previous cohort studies found, that especially small unruptured intracranial aneurysms exhibit a low risk of rupture [4,5,6,7,8]. Aneurysms of the anterior and posterior communicating artery are at higher risk to rupture [6,7]. The major imaging methods for the detection and work up of intracranial aneurysms are computed tomography angiography, magnetic resonance angiography and digital subtraction angiography [9].

Prior to rupture, a cerebral aneurysm might cause symptoms like severe headache, nausea, vision impairment and vomiting [10]. The standard imaging tool for patients reporting to the emergency department constitutes non enhanced computed tomography (NECT) as it is readily available, cheap and fast [11].

Standard NECT images of the neurocranium are reconstructed with slice widths between 5 and 10 mm to reduce image noise resulting in decreased spatial resolution. However, NECT raw data from modern multidetector CT scanners can be reconstructed with small slice width resulting in higher spatial resolution. This technique resulted in a higher sensitivity in the detection of clots occluding proximal intracranial vessels [12].

We hypothesized that the detection rate of aneurysms on thin image slices would similarly be higher and that the higher spatial resolution would outweigh the higher image noise. The aim of our study was to investigate if thin slices image reconstructions of NECT can be used to detect aneurysms in patients that report to the emergency department. These patients should receive further vascular imaging to verify the diagnosis and to evaluate treatment options to prevent future aneurysm related subarachnoid hemorrhage.

## Methods

### Patient selection

All datasets of patients who received time of flight angiography (TOF) and computed tomography angiography (CTA) at the institution of K.M. from November 2010 to December 2015 were reviewed. If an aneurysm was reported, the local PACS database was questioned for prior non-enhanced CT scans (NECT). Patients with signs of major vascular pathology, hemorrhage, tumor or stroke were excluded from the study. Every patient was matched with a control patient who received a NECT scan of the same age and sex without an aneurysm. Control patients with signs of major vascular pathology, hemorrhage tumor and stroke were excluded from the study. In total a collection of 69 NECT datasets with 35 aneurysms were analyzed. One patient had 2 aneurysms, 33 patients had one aneurysm and 35 patients had no aneurysm. The mean and median age of the patients was 59 years with a range between 31 and 76 years. Eighteen patients were females and were 17 male patients. The median time interval between NECT and vascular imaging was 6 days; the mean time interval was 94 days, with a range of 0–1825 days. Eighteen patients received time-of-flight-angiographies and 16 CT-angiographies.

Neither approval of the institutional review board nor patient informed consent were required according to the local ethics committee of the institution due to the retrospective character of the analysis of patient records and imaging. All study protocols and procedures were conducted in accordance with the Declaration of Helsinki. The deidentified data is available from the corresponding author upon reasonable request.

### Data Acquisition

All patients were scanned with a multidetector CT Scanner with 2 × 64 Detector rows (Siemens Somatom Definition, Erlangen Germany). The standard protocol consisted of a collimation of 64 × 0.6 mm, a slide with of 1 mm, a Pitch of 0.55 and a tube voltage of 120 KV. A reconstruction kernel optimized for brain imaging was used. Five-millimeter thick slices were calculated for brain parenchyma imaging as a standard procedure. These images were used to exclude patients with major vascular pathology, tumor or hemorrhage from the study.

### Observational study

**Image 1:**
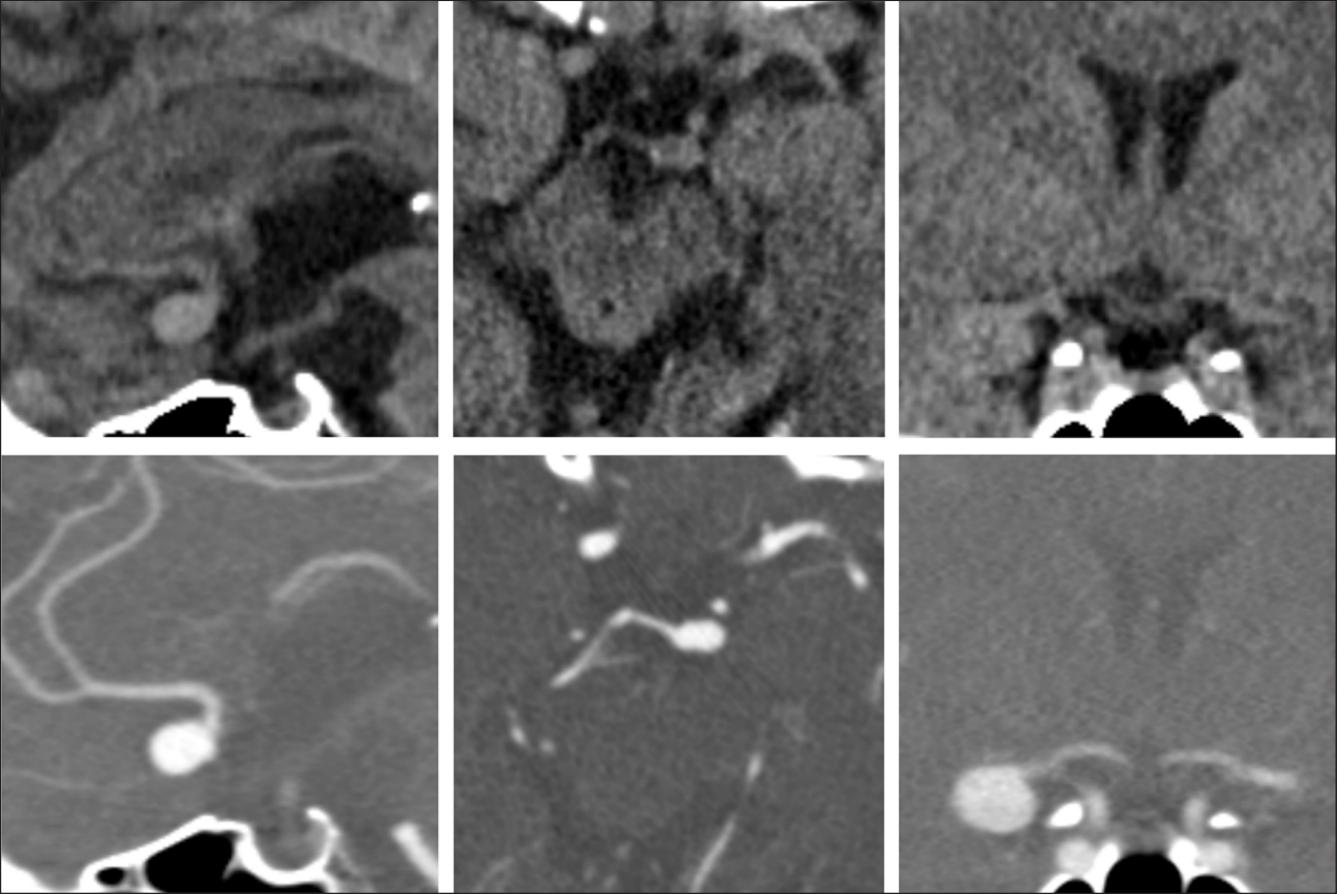
Examples of reconstructed images from the NECT datasets (upper row) and corresponding images from CT angiographies (lower row). Left: sagittal reconstruction of an aneurysm of the anterior communicating artery. Middle: axial reconstruction of an aneurysm of the basilar tip. Right: coronar reconstruction of an aneurysm of the middle cerebral artery.

One experienced neuroradiologist (L.F., seven years of experience) reviewed the randomly arranged NECT datasets regarding the detectability and location of an aneurysm twice with a six-month interval. The reader did not receive the corresponding vascular images and was unaware of the diagnosis. All NECT images were converted into 1.5 mm thick slap maximum intensity projections (MIPs) with an increment of 1 mm in 3 spatial directions. If an aneurysm was suspected on the MIP images, the diagnosis was confirmed using the isotropic raw data set in all 3 spatial directions. The presence and the location of the suspected aneurysm was recorded. The NECT datasets were reviewed and reconstructed on a standard radiological viewing and reporting workstation. Examples for these reconstructed images are displayed in ***Image 1***.

In a second step, all CTA and TOF datasets were reviewed with respect to the prevalence, size and location of an aneurysm on a standard radiological viewing and reporting workstation. The prevalence and size of an aneurysm was confirmed and documented using a standard ruler tool on the isotropic datasets. The measurements and reconstructions were performed with the software Jivex diagnostic client (Visus Health IT, Bochum, Germany).

### Statistical Analysis

The calculations were performed in R version 4.0.3. [13].

A logit regression analysis was performed with size and location as dependent variables.

We used Fisher`s exact test to evaluate the effect of aneurysm size and aneurysm location on the detectability of the aneurysms. The odds ratios for the detection probability and the predicted probability of detection of an aneurysm were calculated for the variable aneurysm size.

For further statistical analysis, 2 × 2 tables were constructed for both passes. The sensitivity, false negative rate, specificity, positive predictive value, false discovery rate, negative predictive value and false omission rate were calculated for both passes and all patients. In a second step, the sensitivity and false negative rate were calculated for aneurysms with 0–6.9 mm, 7–9.9 mm and 10–20 mm. These intervals were chosen according to the intervals used in the calculation of the widely implemented PHASES score [14]. The PHASES score is a practical risk score to aid for the prediction of the risk of rupture of intracranial aneurysms based on a review of six prospective cohort studies with subarachnoid hemorrhage as outcome. Finally, receiver operator characteristics curves and the area under the curve were calculated for both passes.

## Results

In total, 35 aneurysms were analyzed, 8 were located at the internal carotid artery, 10 were located at the anterior communicating artery, 10 were located at the middle cerebral artery, 2 were located at the posterior communicating artery, 4 in the posterior circulation and one at the pericallosal artery. Overall mean size of the analyzed aneurysms was 9 mm (median 8 mm, 1. quartile: 6 mm, 3. quartile: 10 mm. minimum: 3 mm, maximum: 26 mm). When using reconstructed thin slice maximum intensity projections of the NECT, at first 63% of the aneurysms were detected and six months later 66% of the aneurysms were detected. The result of Fisher`s exact test for aneurysm size was a p-value of 0.004 for the second pass and of 0.007 for the first pass. The result of Fisher`s exact test for aneurysm location was a p-value of 0.36 for the second pass and 0.84 for the first pass. The Area under the curve from the Receiver operating characteristics curve analysis was 0.77 for the second pass and 0.78 for the first pass.

For each millimeter increase of aneurysm size, the odds of aneurysm detection increased by a factor of 5.33 for the second pass and by a factor of 3.64 for the first pass. The predicted probabilities from the logit analysis indicate that at the second pass aneurysms >7 mm were detected and at the first pass aneurysms >8 mm were detected.

The results of the 2 × 2 tables are shown in ***Tables 1*** and ***2***.

**Table 1:** Results of the 2 × 2 tables for both passes for the whole studied group.


	SENSITIVITY	FALSE NEGATIVE RATE	SPECIFICITY	POSITIVE PREDICTIVE VALUE	FALSE DISCOVERY RATE	NEGATIVE PREDICTIVE VALUE	FALSE OMISSION RATE

**First pass**	0.66	0.34	0.88	1.29	0.15	0.71	0.28

**Second pass**	0.63	0.37	0.94	1.46	0.08	0.71	0.28


**Table 2:** Results of the 2 × 2 tables for both passes. The study group is subdivided in three classes with different aneurysm sizes. The intervals of the subdivision follow the different size classes of the widely used Phases score.


	FIRST PASS SENSITIVITY	FIRST PASS FALSE NEGATIVE RATE	SECOND PASS SENSITIVITY	SECOND PASS FALSE NEGATIVE RATE

**Size 0–6.9 mm** **(n = 11)**	0.09	0.9	0.027	0.73

**Size 7–9.9 mm** **(n = 10)**	0.8	0.2	0.7	0.3

**Size 10–20 mm** **(n = 12)**	0.92	0.08	0.92	0.08


## Discussion

The main finding in this study is that a relevant number of aneurysms can be identified on thin-slice reconstructions of standard NECT scans using thin slap MIPs. These patients should receive vascular follow up imaging to confirm the diagnosis and to evaluate treatment options to prevent future aneurysm-related subarachnoid hemorrhage.

In the current study, the effect of size on the detection rate of aneurysms was significant for both passes. Aneurysms smaller than 7 mm were detected with a very low sensitivity of 0.09 and 0.03, respectively. In patients with aneurysms of less than 7 mm diameter, without previous subarachnoid hemorrhage, the rupture rate is estimated at around 0.1% per year [8,15]. The sensitivity to detect aneurysms with 7–9.9. mm was 0.8 and 0.7 whereas the sensitivity for aneurysms with 10–20 mm was 0.92 in our study. The Hazard ratio for rupture of aneurysms with 7–9.9 mm was 2.7 and for aneurysms with 10–19.9 mm was 5.3. as compared with aneurysms smaller than 5 mm in a pooled analysis of six prospective cohort studies [14]. The rupture rate rises with the aneurysm size and larger aneurysms that are more prone to rupture were detected with higher sensitivity in our study [6,8,14].

We did not observe a relevant effect of the location on the detectability of an aneurysm, probably due to the small sample size of our study. Aneurysms of the anterior communicating artery and middle cerebral artery exhibit a high inherent contrast between blood filled aneurysm and surrounding liquor filled space. A higher detection rate for smaller aneurysms in this location seems logical. By contrast, aneurysms of the internal carotid artery are obscured by the blood-filled cavernous sinus with a very low inherent contrast. Aneurysms in this location can only be detected by their mass effect with resulting lower detection rate for small aneurysms.

The sensitivity to detect small intracranial aneurysms on thin slice reconstructions of standard NECT is of course much lower than CT-angiography and 3-D-TOF-MRA which are frequently used to detect intracranial aneurysms. As the size of the aneurysm rises, the sensitivity of detecting aneurysms in our study is still lower, but comes closer to CT-angiography and 3-D-TOF-MRA. In two previous studies, the sensitivity of multislice CT was compared to the gold standard 3D Rotational Angiography. The sensitivity to detect intracranial aneurysms smaller than 4 mm was 0.84 and 0.92, for aneurysms with a size between 4 mm and 10 mm the sensitivity ranged between 0.97 and 1 and the sensitivity for aneurysms with a size of over 10 mm was 1 [16,17]. A meta-analysis of studies that investigated 3D TOF Angiographies reported a sensitivity range for the detection of aneurysms smaller than 3 mm between 25% and 99% and for aneurysms larger than 3 mm between 57 and 100% [18].

Small objects like aneurysms or dense clots can be more easily detected on reconstructed CT images with smaller slice width utilizing identical x ray dose [19]. A smaller slice width results in higher spatial resolution and less volume averaging leading to higher object contrast [20]. With respect to the intracranial, cerebral arteries, this gain in contrast outweighs the increased image noise because of the high inherent contrast between blood filled vessel and surrounding subarachnoid, liquor filled space. This inherent contrast is increased when maximum intensity projection images are reconstructed. Our findings are consistent with a previous study, where the reconstruction of maximum intensity projection images resulted in a higher detection rate of dense, occluded vessels in stroke [21].

A major limitation of our study is the small sample size and the single center approach. We utilized a single reader for the presented pilot study and aimed to control for learning effects with a large time interval between the two passes. Larger scale prospective multicenter studies are needed to validate the results of this study. As compared to larger cohorts, aneurysms of the middle cerebral artery in our study are slightly overrepresented and aneurysms of the posterior communicating artery are underrepresented [10]. The effect of aneurysm location on aneurysm detectability needs to be evaluated in larger scale studies.

## Conclusions

NECT as the primary imaging tool in patients that report to the emergency department can be used to detect a significant proportion of intracranial aneurysms larger than 7 mm if the NECT data are properly displayed and reconstructed.
